# Computational study of aggregation mechanism in human lysozyme[D67H]

**DOI:** 10.1371/journal.pone.0176886

**Published:** 2017-05-03

**Authors:** Dharmeshkumar Patel, Serdar Kuyucak

**Affiliations:** School of Physics, University of Sydney, Sydney, New South Wales 2006, Australia; University of Michigan, UNITED STATES

## Abstract

Aggregation of proteins is an undesired phenomena that affects both human health and bioengineered products such as therapeutic proteins. Finding preventative measures could be facilitated by a molecular-level understanding of dimer formation, which is the first step in aggregation. Here we present a molecular dynamics (MD) study of dimer formation propensity in human lysozyme and its D67H variant. Because the latter protein aggregates while the former does not, they offer an ideal system for testing the feasibility of the proposed MD approach which comprises three stages: i) partially unfolded conformers involved in dimer formation are generated via high-temperature MD simulations, ii) potential dimer structures are searched using docking and refined with MD, iii) free energy calculations are performed to find the most stable dimer structure. Our results provide a detailed explanation for how a single mutation (D67H) turns human lysozyme from non-aggregating to an aggregating protein. Conversely, the proposed method can be used to identify the residues causing aggregation in a protein, which can be mutated to prevent it.

## Introduction

Protein aggregation is a fundamental phenomena in molecular biology. It is responsible for the pathogenesis of many diseases, [1, 2] and causes major problems in production and marketing of bioengineered products. [3, 4] Yet we know little about the mechanisms of aggregation at a molecular level. Such knowledge is essential for developing effective therapeutics to treat diseases caused by aggregation, [5–7] and for designing biologics that are aggregation resistant. [8, 9] Computational studies validated by experiments could provide more detailed information about the onset of aggregation. Because of the intense interest on Alzheimer’s disease, initial computational efforts have been mostly focused on formation of amyloid fibrils from the amyloid-*β* peptide. [10, 11] Amyloid-*β* are short (40–42 residues) peptides that form *β*-sheets. Most proteins of pharmaceutical and biotechnological interest have more complex structures and presumably follow a more complicated aggregation process. [3, 4, 8, 9] Early efforts to find aggregation-prone regions in such proteins were based on bioinformatic methods, where the hydrophobic regions in a protein were identified from its sequence and crystal structure. [12–14] This is clearly a very approximate description of protein dynamics and interactions involved in aggregation, and a more realistic description of the aggregation process is desirable.

In a typical scenario, aggregation is initiated by partial unfolding of a protein which then forms a semi-stable dimer. This is followed by formation of multimers and more complex structures. Thus the critical first step in protein aggregation is dimer formation, and its prevention could also help to stop aggregation. This requires a molecular-level understanding of the mechanism of dimer formation, which, in principle, can be obtained from computational studies. Such a study could be performed in three stages: i) finding partially unfolded conformers of a protein which can be facilitated by performing molecular dynamics (MD) simulations at elevated temperatures, [15, 16] ii) searching for the most stable dimers among the conformers using docking methods and refining them in MD simulations, [17, 18] and iii) performing free energy calculations to rank the binding free energies of the dimers and find the most stable complex structure(s). [19, 20] Use of MD simulations is essential in order to obtain accurate complex structures at atomic resolution, which makes such a study computationally quite demanding for large proteins such as therapeutic antibodies. Thus it is prudent to check the viability of this approach first on a small protein.

Human lysozyme (HL) provides an almost ideal test case for this purpose. [21] Two single point mutations in HL (I56T and D67H) cause hereditary systemic amyloidosis, which does not happen in wild type HL. [22] Structural studies showed that both HL[I56T] and HL[D67H] were unstable under physiological conditions, and partly unfolded intermediates were involved in their aggregation. [23, 24] Thus a comparative computational study of dimer formation propensity in one of the mutant HLs versus wild-type HL provides a convenient framework for checking the viability and validity of the proposed approach.

Lysozyme has been widely used as a model system to study how unfolded conformations lead to amyloidosis under various physiological conditions. [25–28] Hen egg white lysozyme has been a popular choice in computational studies of thermal unfolding, where MD simulations were performed at a range of temperatures from 300–500 K to accelerate unfolding. [29–33] There is evidence from computational and experimental studies that proteins unfold following the same pathway at higher temperatures, [34, 35] which rationalizes such an approach. Most of the MD simulations of hen egg white lysozyme were limited in time (1–10 ns), and only in one study trajectories of 1 *μ*s duration were obtained using an IBM Blue Gene supercomputer. [32] Similar computational studies were also performed for HL and its amyloidic variants but the simulations times (1–5 ns) were too short for adequate sampling of the semi-stable conformations involved in aggregation. [36–38] A general conclusion emerging from these MD simulations is that unfolding of lysozyme is initiated by distortions in the three-stranded *β*-sheet domain, which is consistent with experiments. [22, 23]

In the present study, we explore dimer formation in HL[D67H] due to partly unfolded intermediates. Longer MD simulations are performed at both room and higher temperatures to sample such conformers. Potential dimer structures are searched among the set of conformers using the docking program HADDOCK, [39, 40] and the consensus complex structures are further refined in MD simulations. Binding free energy of each complex is estimated using Jarzynski’s equation [41] in steered MD simulations to find the most stable dimer. [42] A parallel study is performed for the wild type HL to provide reference for the HL[D67H] simulations and also to show that the approach is robust enough to distinguish between the aggregation propensity of two proteins that differ by a single mutation.

## Methods

### Structures of human lysozyme and D67H mutant

**Fig 1:**
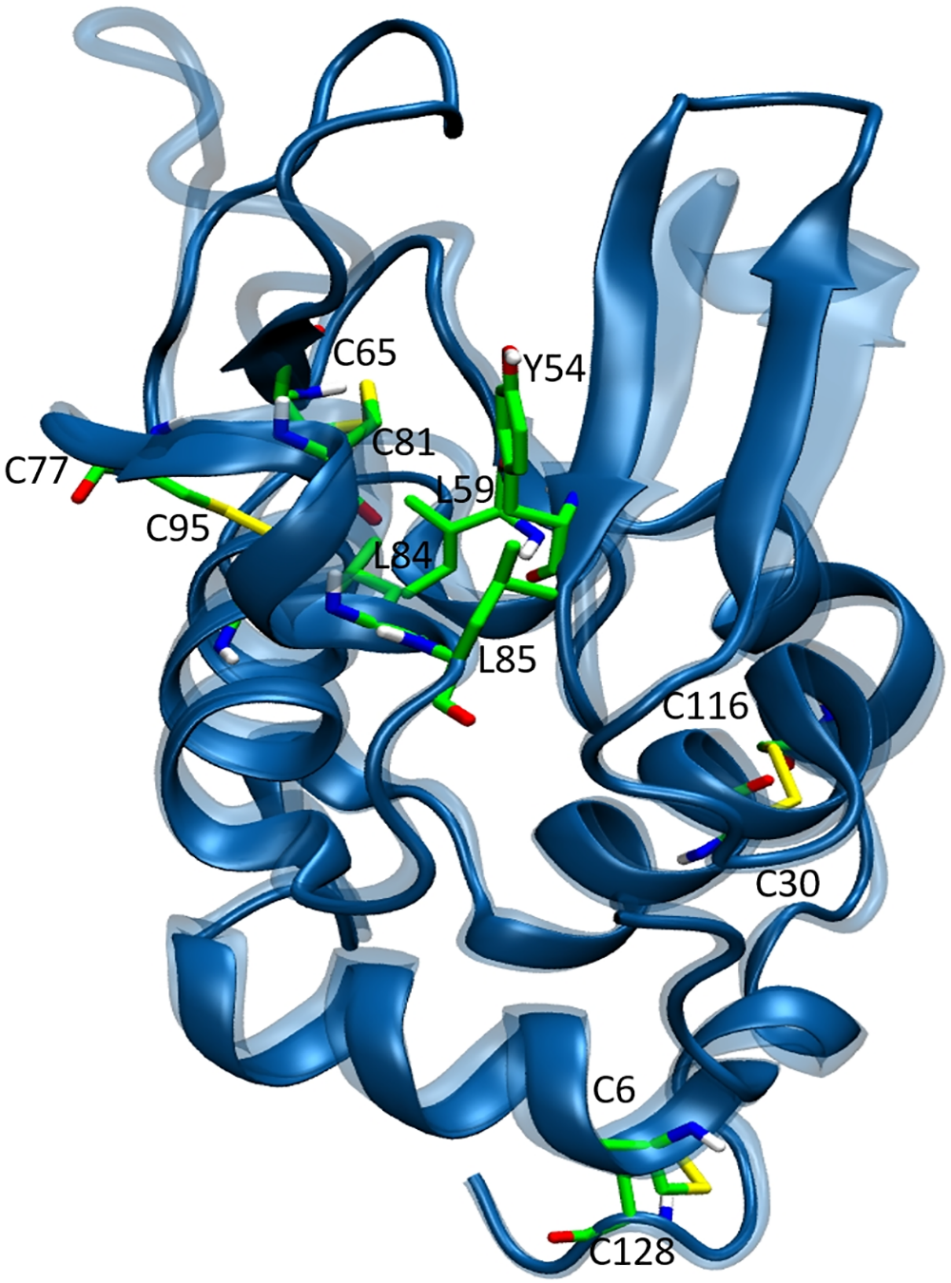
The crystal structure of HL. The side chains of the residues forming the hydrophobic pocket (Y54, I59, C65, C81, L84, and L85) are explicitly shown. The four disulfide bonds are indicated with yellow. The crystal structure of HL[D67H] is superposed on that of HL in translucent color.

**Fig 2:**
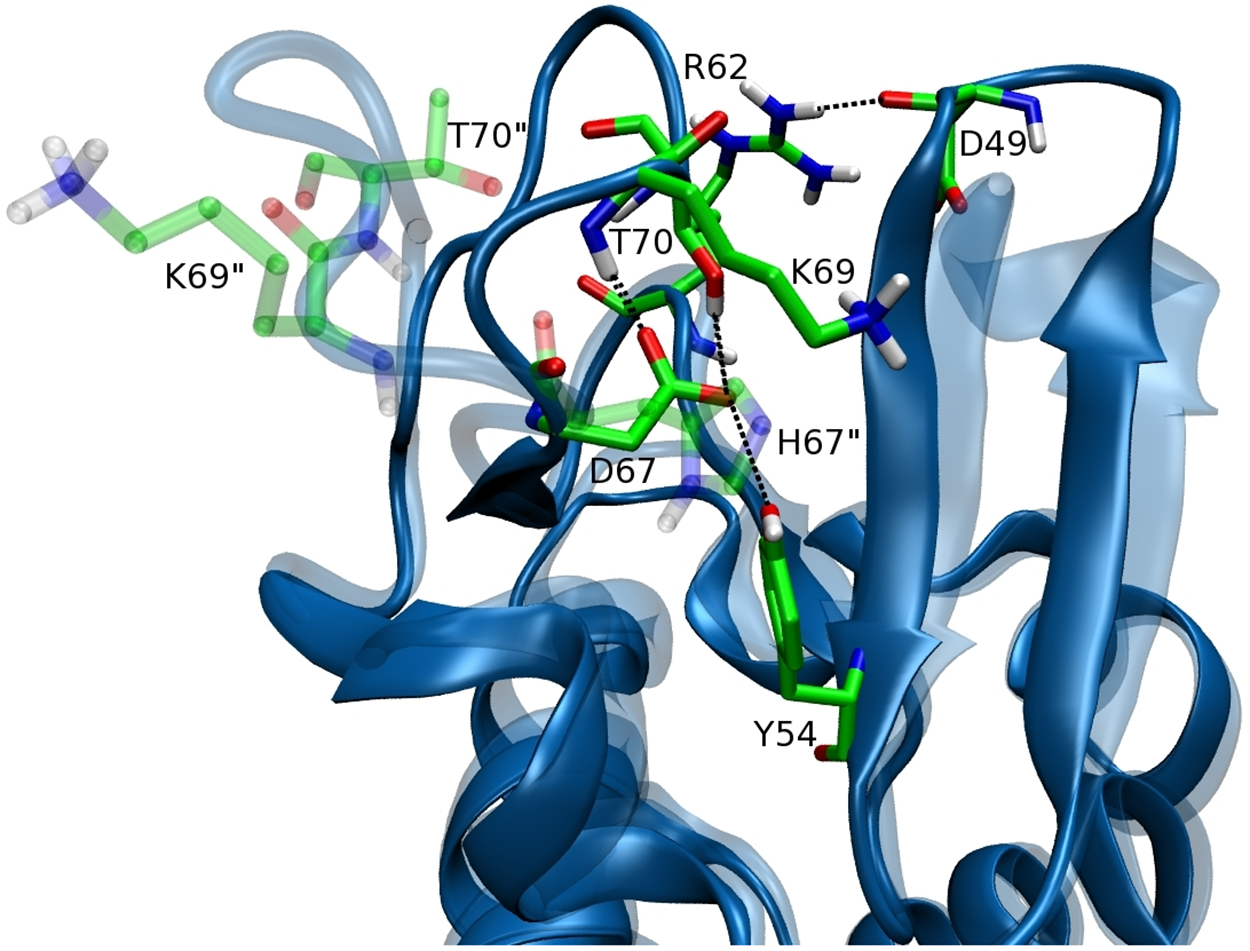
The interactions that protect the hydrophobic pocket in the*β*-domain of HL: D67–{Y54, K69, T70} and R62–D49. The corresponding structure of HL[D67H] is superposed in translucent colors and labeled with double primes where the side chain positions differ.

The crystal structure of HL was determined at 1.5 Å resolution (PDB ID: 1REX, [43] Fig 1). HL is a 130-residue globular protein, consisting of an *α*-domain (residues 1–42 and 81–130) and a *β*-domain (residues (43–80). As both experimental and computational studies indicate that the *β*-domain is involved in unfolding and aggregation of its mutants, [22, 23, 36] we will focus on that region in the following. The HL structure is stabilized by four disulfide bonds between C6–C128, C30–C116, C65–C81, and C77–C95 (Fig 1). The first two are in the *α*-domain while the last two connect the *α* and *β* domains. The *β*-domain starts with a three-stranded antiparallel *β*-sheet (residues 43–60) and continues with a mixture of irregular loops and a *β*-sheet. There is a hydrophobic pocket at the interface of the *α* and *β*-domains formed by the residues Y54, I59, C65, C81, L84, and L85, which plays an important role in unfolding and dimer formation in HL[D67H] (Fig 1). This hydrophobic pocket is protected by two interactions; i) the D67 side chain makes hydrogen bonds with the side chains of Y54 and T70 and also with the backbone amine of K69, and ii) the R62 side chain makes contacts with the side chain and carbonyl oxygens of D49 (Fig 2).

The crystal structure of HL[D67H] (PDB ID: 1LYY [23]) is compared to that of HL in Fig 1. The *α*-domains are seen to overlap well but there are some differences in the *β*-domains, in particular, in the C65–C77 loop (the average backbone RMSD between HL and HL[D67H] is 1.87 Å). This can be traced to the D67H mutation which results in the loss of the hydrogen-bond network mediated by the D67 side chain (Fig 2). Besides movement of the C65–C77 loop away from the *β*-sheet, the K69 side chain also flips away, which further exposes the hydrophobic pocket. But the loop on the other side of C65 is preserved, and the R62–D49 interaction remains intact, which keeps guarding the hydrophobic pocket. We note that the bending of the *β*-turn HL[D67H] relative to that in HL disappears in room temperature MD simulations. So this may not be a genuine feature of the HL[D67H] structure under physiological conditions.

### Molecular dynamics simulations

MD simulations are performed using the NAMD package [44] with CHARMM36 force field. [45, 46] An NpT ensemble is used with periodic boundary conditions. Pressure is fixed at 1 atm while temperature is varied from room temperature (300 K) up to 450 K to induce unfolding in the *β*-domain. The particle-mesh Ewald method is used to evaluate the Coulomb interactions without cut offs while the Lennard-Jones interactions are switched off within 10–12 Å. A time step of 2 fs is used in all MD simulations.

The simulation systems for HL and HL[D67H] are prepared using the VMD software. [47] The crystal structures of both proteins are solvated with at least four layers of water molecules. The systems are ionized and neutralized with 150 mM of NaCl. Initially, the protein atoms are fixed and the systems are equilibrated at 300 K with 1 atm pressure coupling in all directions until the correct water densities are obtained. For HL, the size of the simulation box is 76 x 82 x 76 Å^3^, and it contains 14,080 water molecules. For HL[D67H], the size is 88 x 74 x 78 Å^3^, and it contains 14,862 water molecules. The HL system is simulated at 300 K and 400 K for 100 ns, which provide reference structures for comparison with the HL[D67H] simulations. Systems at elevated temperatures are obtained by slowly heating them at the rate of 10 K/ns.

The choice of 400 K is based on previous MD simulations of HL and hen egg white lysozyme at high temperatures, [29–33, 36–38] and our own test simulations of HL[D67H]. In most MD simulations, the aim is complete denaturation of the protein, so relatively high temperatures (e.g., 500 K) were employed. As we want a limited unfolding that exposes only the hydrophobic pocket in the *β*-domain, we have tried lower temperatures in test simulations. Denaturation of the *α* helices in the *α*-domain is still observed in MD simulations of HL at 450 K but not at 400 K. Further lowering of the temperature to 350 K yields essentially the same results as those at 300 K, and is unlikely to provide the unfolded conformation in a reasonable time frame. Thus 400 K appears to be an optimal choice for achieving the limited unfolding in the *β*-domain.

The trajectory data are saved at every 5 ps to analyze the conformational changes induced on the proteins at high temperatures. Tools used for conformational analysis include the root mean square deviation (RMSD) of the backbone atoms and the residue specific RMSD for global analysis. To gauge the unfolding in the *β*-domain that leads to the exposure of the hydrophobic pocket, we use the time series of the distances between the interacting residues shown in Fig 2 and the number of water molecules occupying this pocket.

### Molecular docking

After identifying the stable structures of HL and HL[D67H] at both room and high temperatures, we have searched for potential dimer structures among this set using the docking program HADDOCK. [39, 40] We have previously used HADDOCK in several studies of toxin binding to ion channels and found that the binding poses obtained from HADDOCK required minimal refinement of the complex structure in subsequent MD simulations. [48–50] To avoid any biasing, blind docking is performed initially for a given pair of monomers. Potential restraints identified from cluster analysis of the initial docking results are employed in a second stage of docking to improve the binding pose and ensure its statistical reliability. In the last stage, consensus docking poses are refined in MD simulations performed at 300 K. The complex structures are equilibrated using the protocols mentioned above, followed by production runs for up to 100 ns. The results of the MD simulation are used to check the stability of the complex structures. Those that have dissociated during the MD simulations are discarded while the ones that have remained stable are subjected to free energy calculations to find their ranking.

### Free energy profiles

The purpose of the free energy calculations is to find the most stable dimer structure(s) that may be involved in the aggregation of HL[D67H]. Accuracy in ranking rather than the absolute free energies is sufficient for this purpose. Therefore, we use the steered MD with Jarzynski’s equality to estimate the free energy profiles for dissociation of the dimers. [41, 42] We note that alternative methods such as umbrella sampling MD simulations could provide more accurate free energies but are difficult to implement for large ligands. In steered MD, a harmonic force is applied to the center of mass of one of the biomolecules in the docked complex via a stiff spring, whose reference point is pulled along the reaction coordinate at a constant velocity *v*,

$$\begin{array}{c} z_{\text{ref}}\left(t\right)=z_{0}+vt. \end{array}$$ (1)

Here the reaction coordinate is taken along the *z* axis, and *z*_0_ refers to the initial position of the biomolecule pulled from the binding site. For each simulation path, the work done *W*(*z*) is calculated from the integral of the force on the spring as a function of *z*. The free energy profile along the reaction path, Δ*G*(*z*), is determined from the ensemble average of the work done for many paths using Jarzynski’s equation [41]

$$\begin{array}{c} e^{-\Delta G(z)/\text{kT}}=\left\langle e^{-W(z)/\text{kT}}\right\rangle \end{array}$$ (2)

Steered MD simulations are performed at 300 K. For the spring constant, *k*_s_ = 50 kcal/mol/Å^2^ is used, which is appropriate for stiff spring approximation. Earlier tests have suggested that slower pulling velocities are more likely to improve free energy profiles compared to more sampling. [42] Therefore, the pulling velocity is taken as *v* = 1 Å/ns, which is much smaller than the typical velocities used in steered MD simulations, but only ten simulations are performed for each free energy profile. The starting configurations are generated from the MD simulations of the equilibrated complex structure at 1 ns intervals. The pulling is continued until the ligand reaches the bulk environment, which occurs at about 15 Å from the initial position.

## Results and discussion

### Analysis of conformational changes in HL

**Fig 3:**
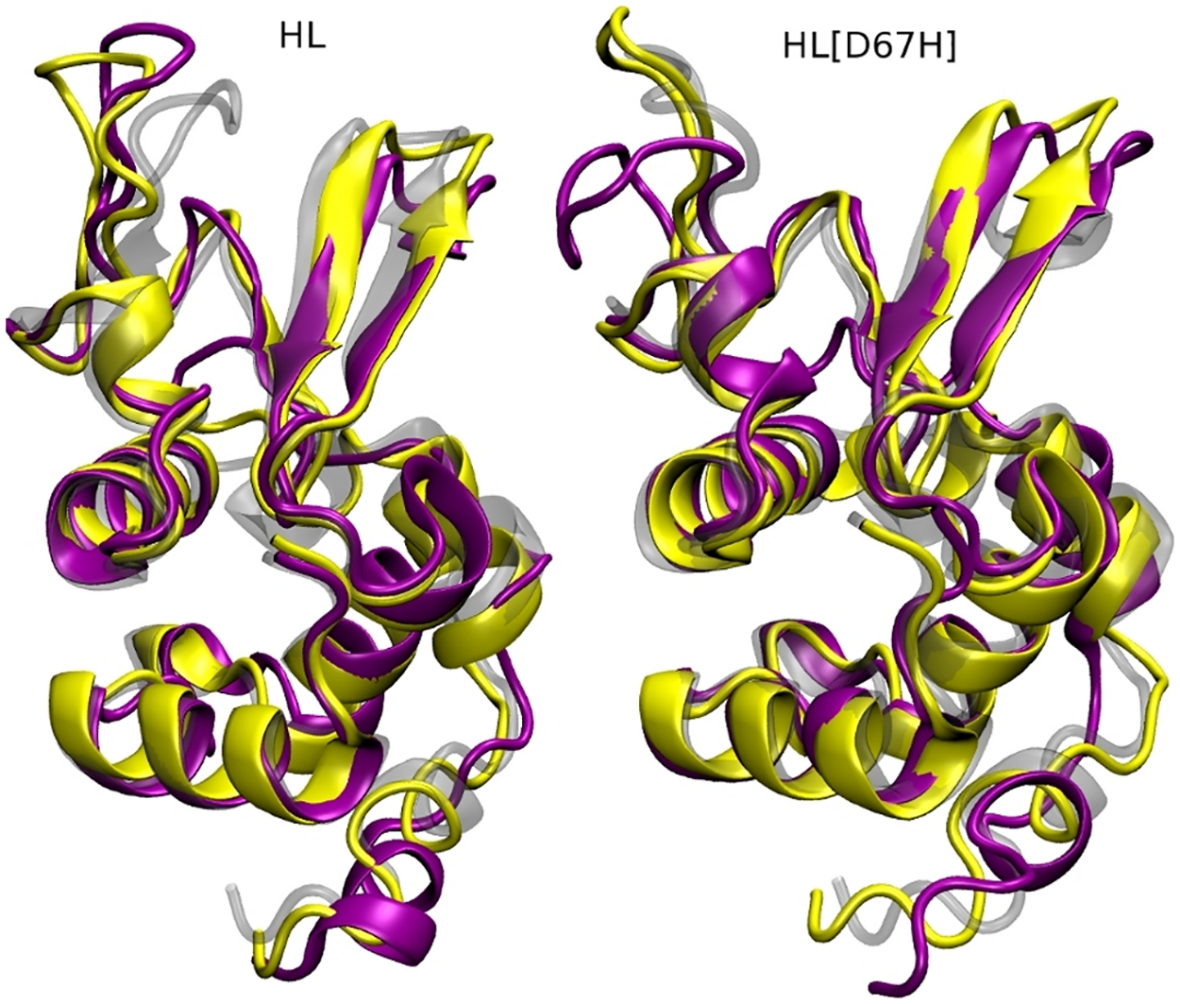
Comparison of the crystal structures (transparent gray) of HL (left) and HL[D67H] (right) with the snapshots obtained from the MD simulations at 300 K (yellow) and 400 K (purple).

**Fig 4:**
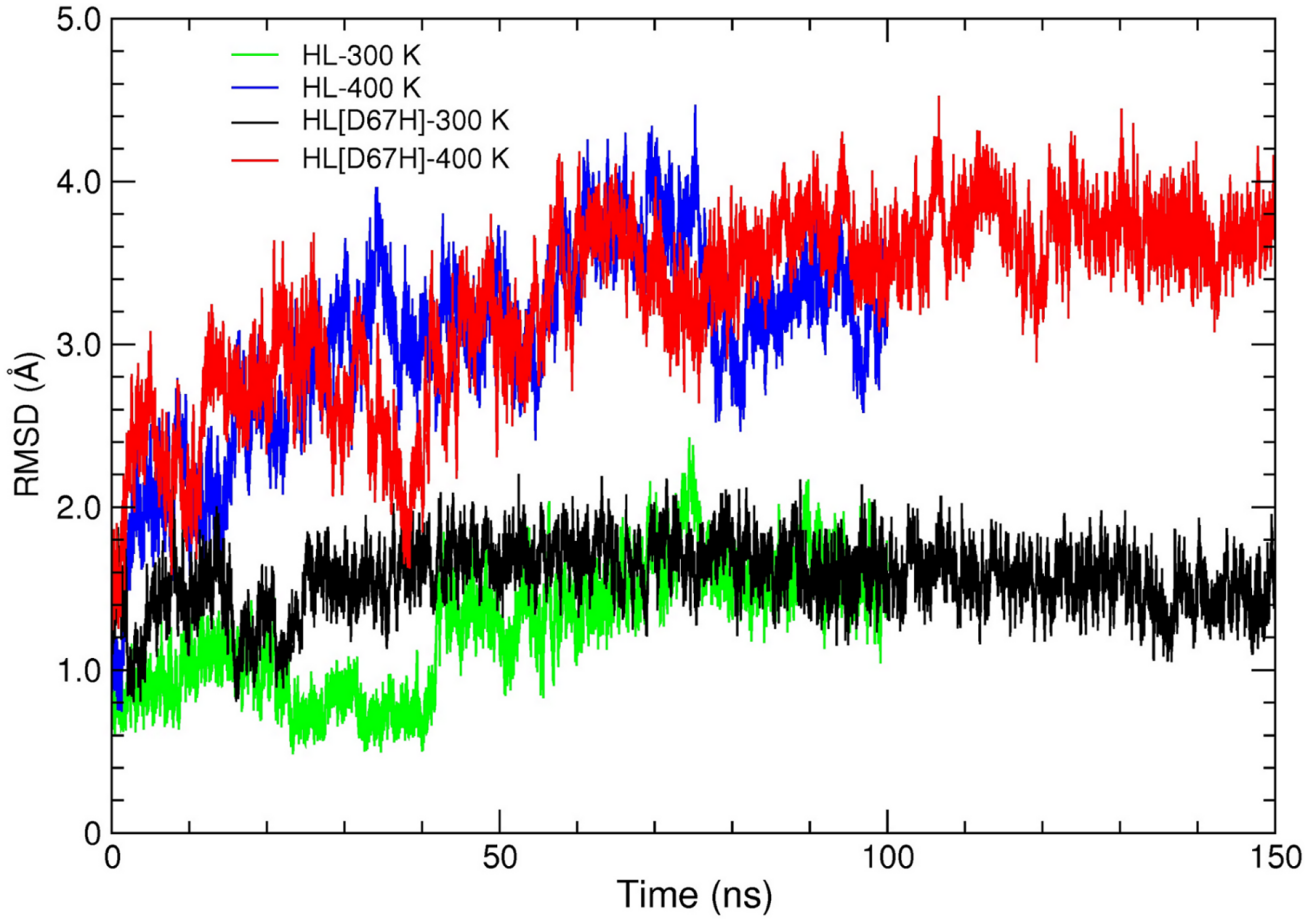
RMSD of the backbone atoms of HL and HL[D67H] at 300 K and 400 K plotted as a function of time. The crystal structure of HL is used as reference in all cases in order to facilitate comparison.

**Fig 5:**
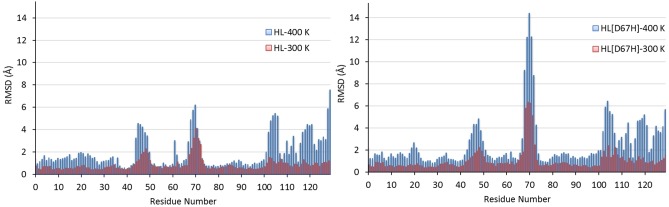
Comparison of the residue specific average RMSDs of the C*α* atoms at 300 K and 400 K for HL (left) and HL[D67H] (right). The crystal structure of HL is used as reference in all cases. The average RMSDs are obtained from the last 25 ns of the trajectory data for HL and from the last 50 ns for HL[D67H].

We first discuss the results of the MD simulations for HL at 300 K and 400 K, which provide reference structures for those of HL[D67H]. To give a broad view of the simulation results, we compare the final snapshots of HL obtained from the 300 K and 400 K simulations with the crystal structure (Fig 3). It is seen that the secondary structure of HL is well preserved at 400 K. The effect of high temperature is mainly confined to the loop regions (e.g., the C65–C77 loop and the *β*-turn), which already exhibit deviations from the crystal structure at 300 K due to fluctuations. In Fig 4, we show the evolution of the backbone RMSDs at 300 K and 400 K. The RMSDs at 400 K are substantially larger than those at 300 K, indicating that permanent deviations from the crystal structure have occurred in some regions. To identify those regions, we compare the average RMSDs of the C*α* atoms at 300 K and 400 K (Fig 5). In several locations, the C*α* RMSDs are above average at 300 K and rise substantially at 400 K. The first peak in HL RMSDs (residues 45–51) corresponds to the *β*-turn and the second peak (residues 68–73) is associated with the C65–C77 loop in the *β*-domain. There are several peaks in the *α*-domain after the residue P103. This region consists of short *α*-helices connected by loops and envelopes the *α*-domain. The disulfide bond at C116 provides some stability but otherwise the whole region remains quite flexible. Thus all the large changes in RMSDs are confined to the loop regions.

Preservation of the secondary structures of HL at 400 K is necessary but not sufficient to ensure that this temperature can be safely used to induce partial unfolding of HL[D67H]. We also need to show that the large fluctuations of the loops in the *β*-domain do not result in exposure of the hydrophobic pocket in HL. As discussed in the Methods, the hydrophobic pocket is protected by several interactions. To check the stability of these interactions, we inspect the time series of the D67–Y54 and R62–D49 distances from the MD simulations at 300 K and 400 K (Fig A in S1 File). At 300 K, the N–O distance between the side chains of R62 and D49 is well preserved thanks to the ionic bond. The O–O distance between the side chains of D67 and Y54 is also maintained throughout the simulations, though it exhibits more fluctuations due to the weaker interaction. Increasing the temperature to 400 K results in larger fluctuations in the D67–Y54 distance but the contact is still preserved. The R62–D49 distance exhibits even larger fluctuations and completely breaks around 75 ns, after which the R62 side chain makes a link with the carbonyl oxygen of T70. Formation of the R62–T70 link prevents opening of the C65–C77 loop, which will be seen as the precursor for the exposure of the hydrophobic pocket. Thus, despite the loss of the R62–D49 ionic bond at 400 K, the hydrophobic pocket is still protected by the D67–Y54 and R62–T70 interactions, and is not exposed.

### Analysis of conformational changes in HL[D67H]

We next discuss the MD simulations of HL[D67H] at 300 K and 400 K using the HL results as reference. Comparison of the final snapshots from the HL[D67H] simulations with the crystal structure reveals a similar picture to that found in HL; the secondary structure is well preserved at 400 K and some changes occur in the loop regions (Fig 3). A notable difference from HL is that larger deviations occur in the C65–C77 loop and the hydrophobic pocket. The backbone RMSDs in HL[D67H] at 300 K and 400 K are also very similar to those in HL after equilibration (Fig 4), confirming that the two proteins have comparable conformations. The slightly larger RMSDs in HL[D67H] at 400 K can be explained by the larger deviations in the C65–C77 loop. This observation is quantified by comparing the average RMSDs of the C*α* atoms in HL[D67H] with those in HL (Fig 5). Overall, there is very good correspondence between the RMSDs of HL[D67H] and HL. The locations of the peaks and their values match quite well at both 300 K and 400 K, except for the peak at the residues 68–73. The peak value in HL[D67H] at 300 K is 50% larger than that of HL at 300 K and similar to the value of HL at 400 K, suggesting that the hydrophobic pocket is not breached in the 300 K MD simulations of HL[D67H]. The enhancement factor between HL[D67H] and HL becomes 150% at 400 K, which is large enough to expose the hydrophobic pocket.

The analysis of the interactions in HL has shown the important role played by D67 in preventing unfolding in the *β*-domain. In the HL[D67H] mutant, H67 does not form any links with the neighboring residues, thus we expect unfolding in the *β*-domain to occur once the R62–D49 ionic bond is broken. Inspection of the time series of the R62–D49 distance obtained from the 300 K MD simulations of HL[D67H] indicates that this has not occurred at room temperature (Fig B in S1 File). Increasing the temperature to 400 K, facilitates the breaking of the R62–D49 ionic bond which occurs at about 100 ns. After the breaking, the R62–D49 distances remain smaller in HL[D67H] compared to HL, which may appear surprising. This happens because the R62 side chain in HL[D67H] cannot make a link with T70 on the C65–C77 loop, which moves even further away from the *β*-turn at 400 K.

**Fig 6:**
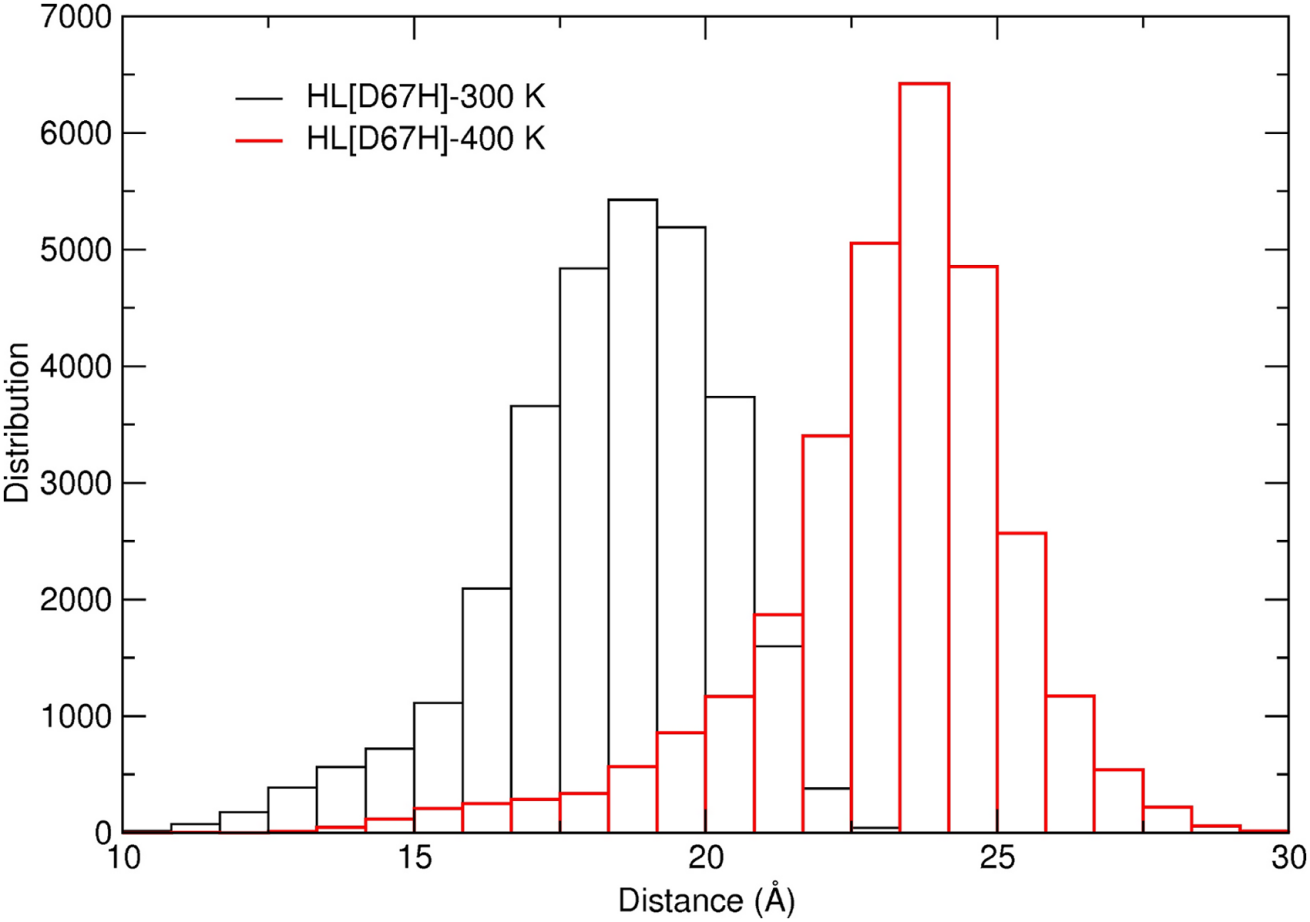
Water molecules (red) in the hydrophobic pocket of HL and HL[D67H] at 300 K and 400 K. Water molecules within 6 Å of the S atom of C65 (indicated with a *) are shown. Side chains of the hydrophobic residues forming the pocket are also shown (green).

So far we have discussed the structural changes that occur in HL[D67H] when the temperature is raised from 300 K to 400 K, but have not provided concrete evidence for the exposure of the hydrophobic pocket. This is difficult to show by just superposing the snapshots as done in Fig 3. We use instead the number of water molecules in the hydrophobic pocket for this purpose. The C65–C81 disulfide bond is at the base of the pocket. Thus we use a sphere centered at the S atom of C65 to determine the average number of water molecules in the pocket. To find the optimal value of the radius, we have tried several radii from 3–6 Å (Table 1). Increasing the radius further resulted in nonzero values for HL at 300 K, indicating that bulk water are counted, so we choose 6 Å as the optimal radius. Inspection of Table 1 shows that HL at 400 K or HL[D67H] at 300 K have no water within 5 Å, and only allow a peripheral water at 6 Å (Fig 6). This demonstrates that the hydrophobic pocket is not breached in MD simulations of HL at 400 K or HL[D67H] at 300 K. In contrast, HL[D67H] at 400 K has water in every shell from 3–6 Å, indicating that the pocket is filled with water (Fig 6).

**Table 1:** Number of water molecules in the hydrophobic pocket of HL and HL[D67H].

Radius	HL[D67H]	HL[D67H]	HL	HL
(Å)	400 K	300 K	400 K	300 K
3	1.3 ± 0.1	0	0	0
4	2.4 ± 0.1	0	0	0
5	5.2 ± 0.5	0	0	0
6	6.4 ± 1.1	1.1 ± 0.4	1.2 ± 0.3	0

The number of water molecules in a sphere centered at the S atom of C65. The radius of the sphere is varied from 3–5 Å. The average values (rounded up to the nearest integer) are obtained from the last 25 ns of the trajectory data for HL and from the last 50 ns for HL[D67H].

**Fig 7:**
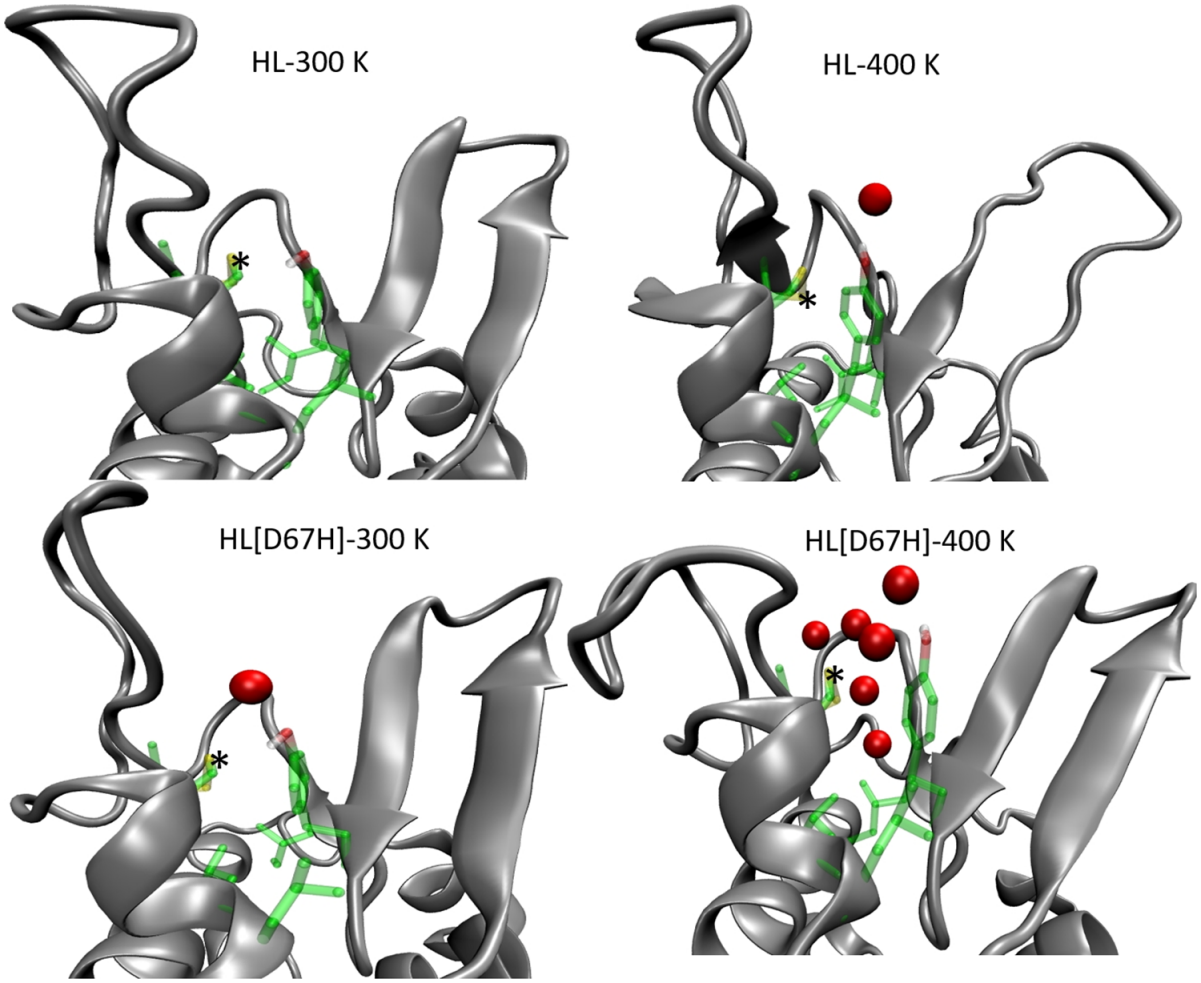
Histogram analysis of the distance between the C*α* atoms of T70 and D49 obtained from the MD simulations of HL[D67H] at 300 K and 400 K. The last 50 ns of the trajectory data are used in the analysis.

Comparison of the HL and HL[D67H] simulations at 300 K and 400 K shows that exposure of the hydrophobic pocket is closely correlated with the opening of the C65–C77 loop away from the *β*-turn (see also Fig 6). Thus a robust measure of the unfolding in the *β*-domain can be obtained from the distance between the C*α* atoms of T70 and D49 (Fig 2). The average T70–D49 distance in HL is 14.0 Å at 300 K and 18.2 Å at 400 K. The corresponding values in HL[D67H] are 18.3 Å at 300 K and 23.5 Å at 400 K. This suggests a threshold T70–D49 distance of > 20 Å for inducing the conformational change to the partially unfolded state. To get more information on these conformations, we perform a histogram analysis of the T70–D49 distance for HL[D67H] at 300 K and 400 K (Fig 7). Both distributions are approximately Gaussian, indicating that both conformations are properly sampled. More importantly, both the folded and unfolded states in HL[D67H] can be adequately represented by a single structure chosen from the most commonly occurring structures in the histogram. This makes the search for potential dimer structures relatively easier as the number of such structures grows quadratically with the number of distinct monomer conformations.

### Search for dimer structures

We have searched for dimer structures among the two HL[D67] conformers using the docking program HADDOCK as described in Methods. The results obtained from three docking studies are as follows.

i) Docking of HL[D67H]–400 K with itself: From clustering analysis of top 100 poses, three distinct complex structures are identified. These are called Complex-1, Complex-2 and Complex-3, following their ranking from energy scores. Inspection of the snapshots of the three poses (Fig C in S1 File) shows that the binding interface is formed by the unfolded parts of the *β* domains in each case. In particular, the C65–C77 loop is seen to play a prominent role in the binding modes.

ii) Docking of HL[D67H]–400 K with HL[D67H]–300 K: Two distinct complex structures are found from clustering analysis in this case, called Complex-4 and Complex-5. The binding interface again involves the C65–C77 loop and the *β*-turn but there is less surface contact, especially in Complex-5 (Fig D in S1 File).

iii) Docking of HL[D67H]–300 K with itself: No docking poses are found in this case, consistent with the hypothesis that partial unfolding triggers dimerization.

Similar docking studies are performed for the HL–300 K and HL–400 K conformers. Only in the case of HL–400 K docking with itself, a binding pose was found (called Complex-6). The binding interface of Complex-6 is very different from the previous complexes and does not involve the C65–C77 loop at all (Fig D in S1 File). HL–400 K and HL[D67H]–300 K conformers look similar with regard to the opening of the *β* domain (e.g., Table 1 and Fig 6) so it may appear surprising that a binding pose is not found in the latter case. Comparison of the residue-specific RMSDs for the two cases (Fig 5) shows that the deviations in the *β*-turn (residues 45–51) are much larger in HL–400 K than HL[D67H]–300 K. Because the *β*-turn is involved in the binding interface of HL–400 K dimer (Fig D in S1 File), this could explain the difference between the two cases.

**Fig 8:**
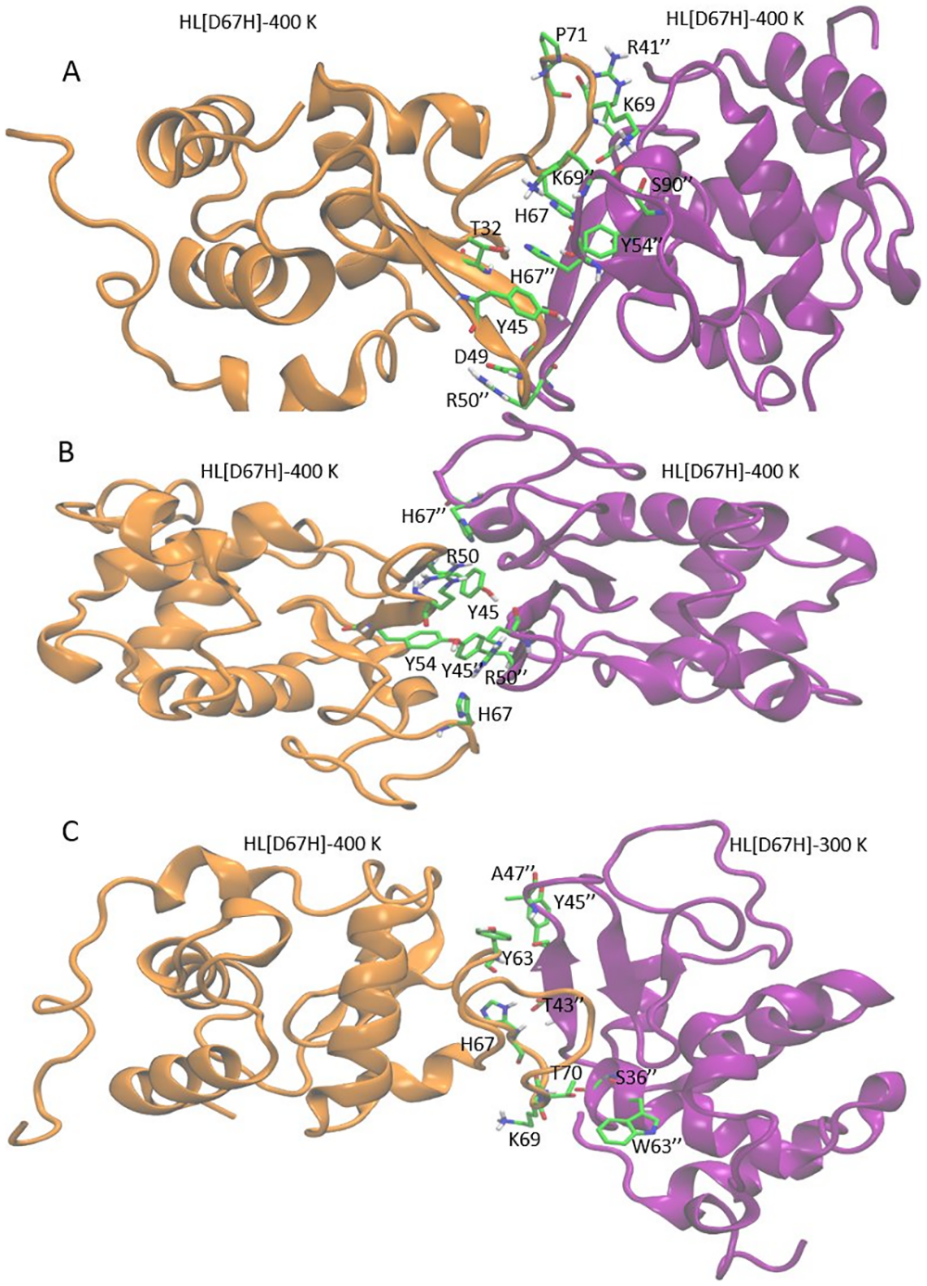
Snapshots of the stable binding modes obtained from the MD simulations for (A) Complex-1, (B) Complex-2, and (C) Complex-4. The side chains involved in the binding are shown explicitly (labeled with double prime in monomer-2 on the right).

In the next step, the six complex structures obtained from docking are refined in MD simulations lasting up to 100 ns. The stability of the dimer structures are monitored by plotting the center of mass to center of mass distance between the two monomers as a function of time (Fig E in S1 File). The monomers in dimers are observed to dissociate in three cases; Complex-6 at 28 ns, Complex-5 at 60 ns, and Complex-3 at 70 ns. We note that Complex-3 and Complex 5 are the lowest ranking structures identified from the clustering analysis, and Complex-6 does not exhibit any strong interactions at the binding interface. The MD simulations for these complexes are terminated once the monomers have separated more than 10 Å. The other three dimers have remained bound throughout the MD simulations (Fig E in S1 File). The snapshots of the final structures for Complex-1, 2, and 4 are shown in Fig 8. The residues involved in the binding are indicated explicitly. The most prominent feature of the binding mode in Complex-1 is the strong pi-stacking interactions among the residues H67–H67″–Y45 and H67–Y54″, which have been exposed after the opening of the *β*-domain. Time series of the distances between the centers of the aromatic rings for these three interactions show the stability of the pi-stacking (Fig F in S1 File). There are also several charge interactions both above and below the pi-stacking, which fortify the binding mode. In Complex-2, hydrophobic interactions occur between Y45–H67″ and H67–Y45″, reflecting the symmetric nature of the binding mode. The number of charge interactions in Complex-2 is also reduced—only two are present. In Complex-4, the number and quality of contacts in the binding mode are further reduced. Thus, from the comparison of the binding modes, we expect Complex-1 to have the highest affinity, followed by Complex-2 and Complex-4.

### Free enery profiles

**Fig 9:**
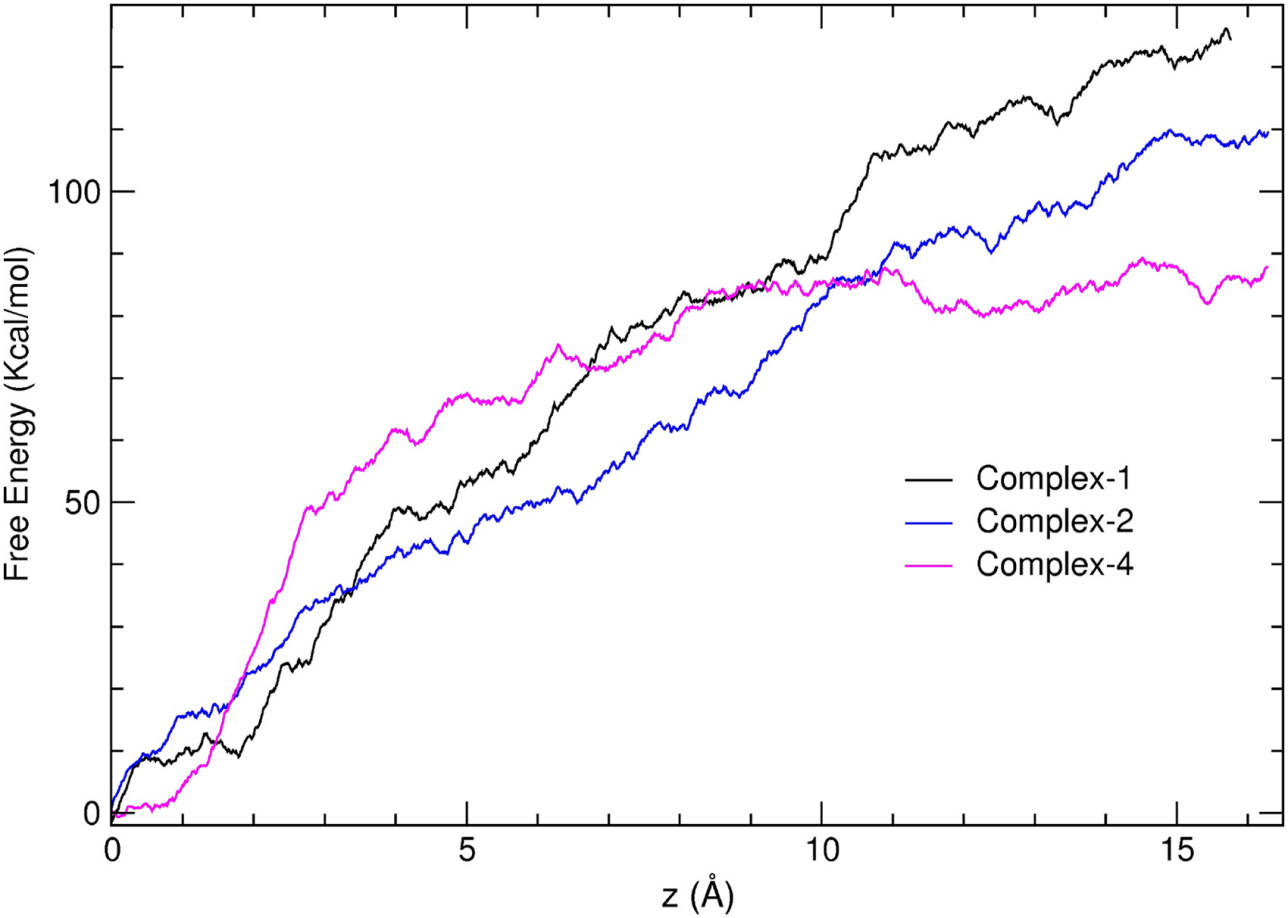
Free energy profiles for dissocation of Complex-1, 2, and 4 obtained from steered MD simulations using Jarzynski’s equation. Complex-1 has the highest affinity, and therefore predicted to be the most stable dimer.

Comparing the number of contacts allows a qualitative ranking of the complexes. For a more quantitative prediction of the ranking, we need to estimate the binding free energies of the three stable complexes. We use the steered MD with Jarzynski’s equation for this purpose as described in Methods. The free energy profiles (Fig 9) show that Complex-1 has the highest affinity followed by Complex-2 and Complex-4, consistent with the ranking of affinities indicated by the binding modes. Thus we predict Complex-1 to be the most stable dimer configuration that provides the initial seed for aggregation of HL[D67H].

We note that the free energy profiles are overestimated using this method, and the magnitude of error grows with the size of the ligand. [42] Because lysozyme is a much larger ligand compared to those used in earlier steered MD calculations, [42] the errors arising from the use of the nonequilibrium processes are more substantial for the lysozyme dimer. To give an idea about the size of such errors, we have repeated the same calculation for Complex-6 at the point of dissociation (Fig E in S1 File) and obtained 50 kcal/mol for the affinity. Nevertheless, such errors are not expected to affect the ranking predicted in Fig 9 because the complexes have similar binding interfaces, and therefore their free energy profiles should also have similar errors.

## Conclusions

We have performed a proof of concept study of dimer formation propensity in HL and HL[D67H] using MD simulations and shown that stable dimer structures form only in the latter case, consistent with the experimental observations. While this has yielded a molecular-level explanation of how HL[D67H] dimers are formed, the real outcome of this study is to propose a method for finding the aggregation prone regions of proteins, and preventing it through suitable mutations. Assuming that protein XYZ aggregates through dimerization of partially unfolded conformers, the method for finding analogs that don’t aggregate consists of the steps: i) perform high-temperature MD simulations of XYZ (together with room temperature simulations for reference) to find the partially unfolded conformers involved in dimer formation ii) dock the conformers and refine them in MD simulations to find the most stable dimer structures, iii) perform free energy calculations to rank the dimers, iv) study the binding mode of the most stable dimer and the corresponding partially unfolded monomer to identify the residues whose mutation could reduce the dimer affinity and/or prevent the unfolding of XYZ. In the case of HL, the D67H mutation is seen to play a dual role—it facilitates unfolding as well as playing a key role in the dimer formation. This explains how a single mutation can have such a drastic effect on the aggregation propensity of HL.

Aggregation of proteins is a major problem in human health and production of pharmaceutical and bioengineered products. Rational solution of this problem requires a molecular-level understanding of the interactions that trigger aggregation. We have described such a method here based on MD simulations, which should help in the design of aggregation-resistant proteins. We hope to apply it to therapeutic proteins such as monoclonal antibodies, where there is a great demand for aggregation-resistant products.

## Supporting information

S1 File S1 File contains 6 figures. **Fig A**, Time series of the D67–Y54 (top) and R62–D49 (bottom) distances in HL at 300 K and 400 K. **Fig B**, Time series of the R62–D49 distance in HL[D67H] at 300 K and 400 K. **Fig C**, Binding modes of HL[D67H] dimers obtained from blind docking of the structures at 400 K. **Fig D**, Binding modes of HL[D67H] dimers obtained from blind docking of the structure at 400 K with a structure at 300 K (complex-4 and 5). Binding mode of HL dimer obtained from blind docking of the structures at 400 K (complex-6). **Fig E**, Time series of the center of mass to center of mass distance between the two monomers obtained from the MD simulations of the six complexes. Complex-1, 2, and 4 remain stably bound whereas Complex-3, 5, and 6 dissociate. **Fig F**, Time series of the distances between the centers of the aromatic rings for the pi-stacking interactions between H67–H67″, Y45–H67″, and H67–Y54″ in Complex-1.
(PDF)
