# Supplementary material for: Computational study of aggregation mechanism in human lysozyme[D67H]
Source: PLoS One. 2017 May 3;12(5):e0176886. doi: 10.1371/journal.pone.0176886 (PMC5415109; doi:10.1371/journal.pone.0176886)
Supplement: S1 File — Fig A, Time series of the D67–Y54 (top) and R62–D49 (bottom) distances in HL at 300 K and 400 K. Fig B, Time series of the R62–D49 distance in HL[D67H] at 300 K and 400 K. Fig C, Binding modes of HL[D67H] dimers obtained from blind docking of the structures at 400 K. Fig D, Binding modes of HL[D67H] dimers obtained from blind docking of the structure at 400 K with a structure at 300 K (complex-4 and 5). Binding mode of HL dimer obtained from blind docking of the structures at 400 K (complex-6). Fig E, Time series of the center of mass to center of mass distance between the two monomers obtained from the MD simulations of the six complexes. Complex-1, 2, and 4 remain stably bound whereas Complex-3, 5, and 6 dissociate. Fig F, Time series of the distances between the centers of the aromatic rings for the pi-stacking interactions between H67–H67″, Y45–H67″, and H67–Y54″ in Complex-1. (PDF) [file pone.0176886.s001.pdf]

Supporting Information S1 File

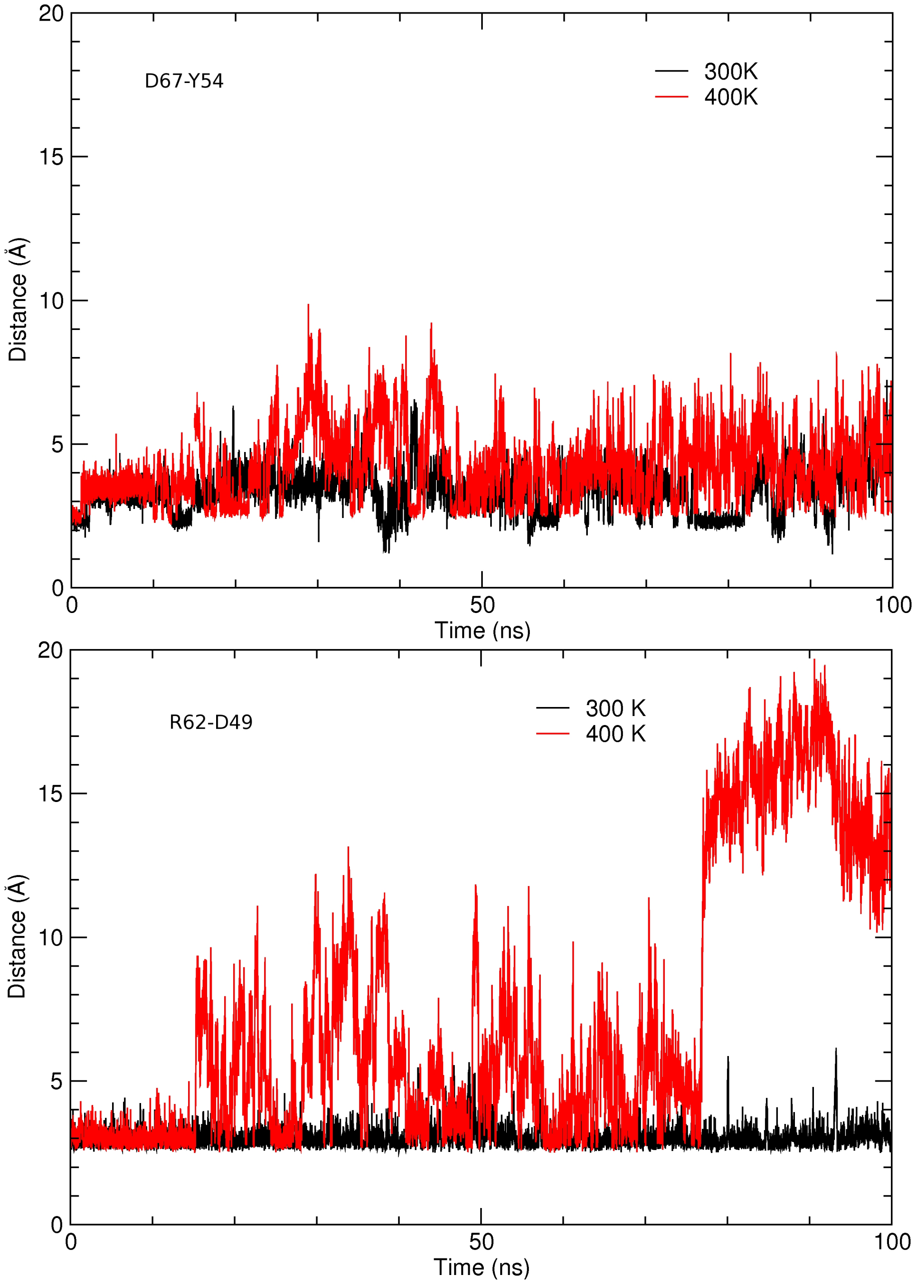

Figure A: Time series of the D67–Y54 (top) and R62–D49 (bottom) distances in HL at 300 K and 400 K.

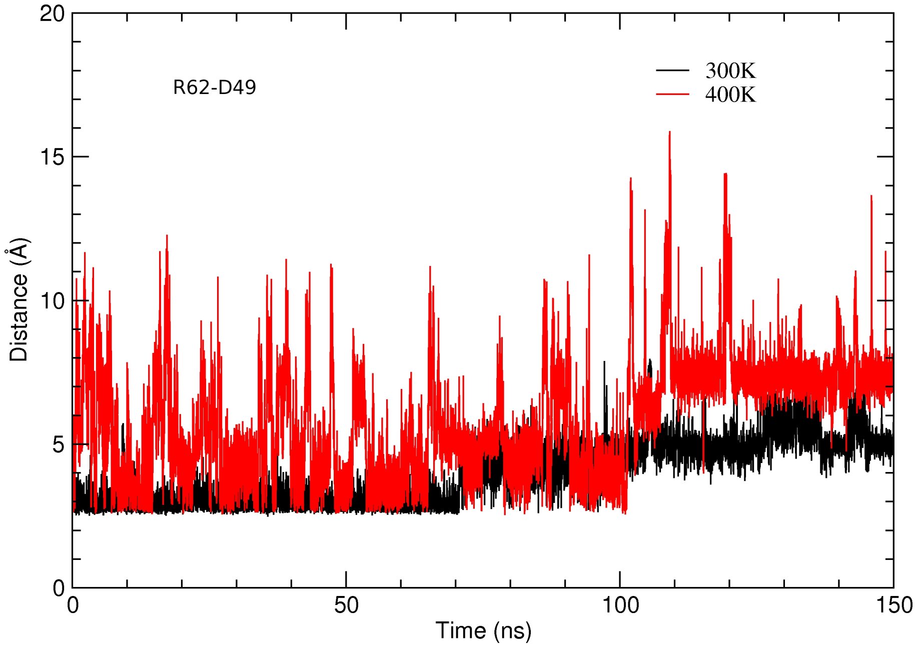

Figure B: Time series of the R62–D49 distance in HL[D67H] at 300 K and 400 K.

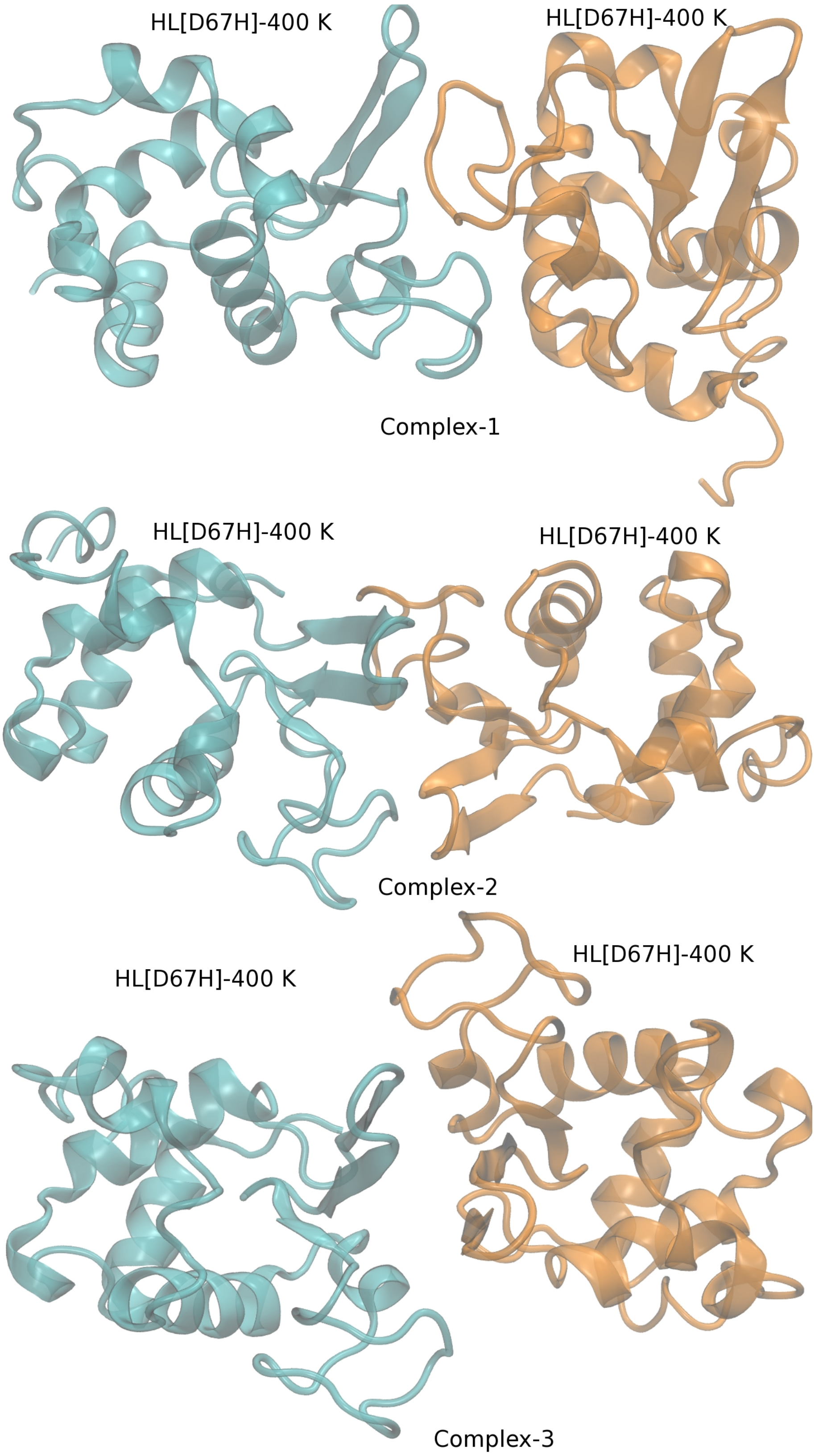

Figure C: Binding modes of HL[D67H] dimers obtained from blind docking of the structures at 400 K.

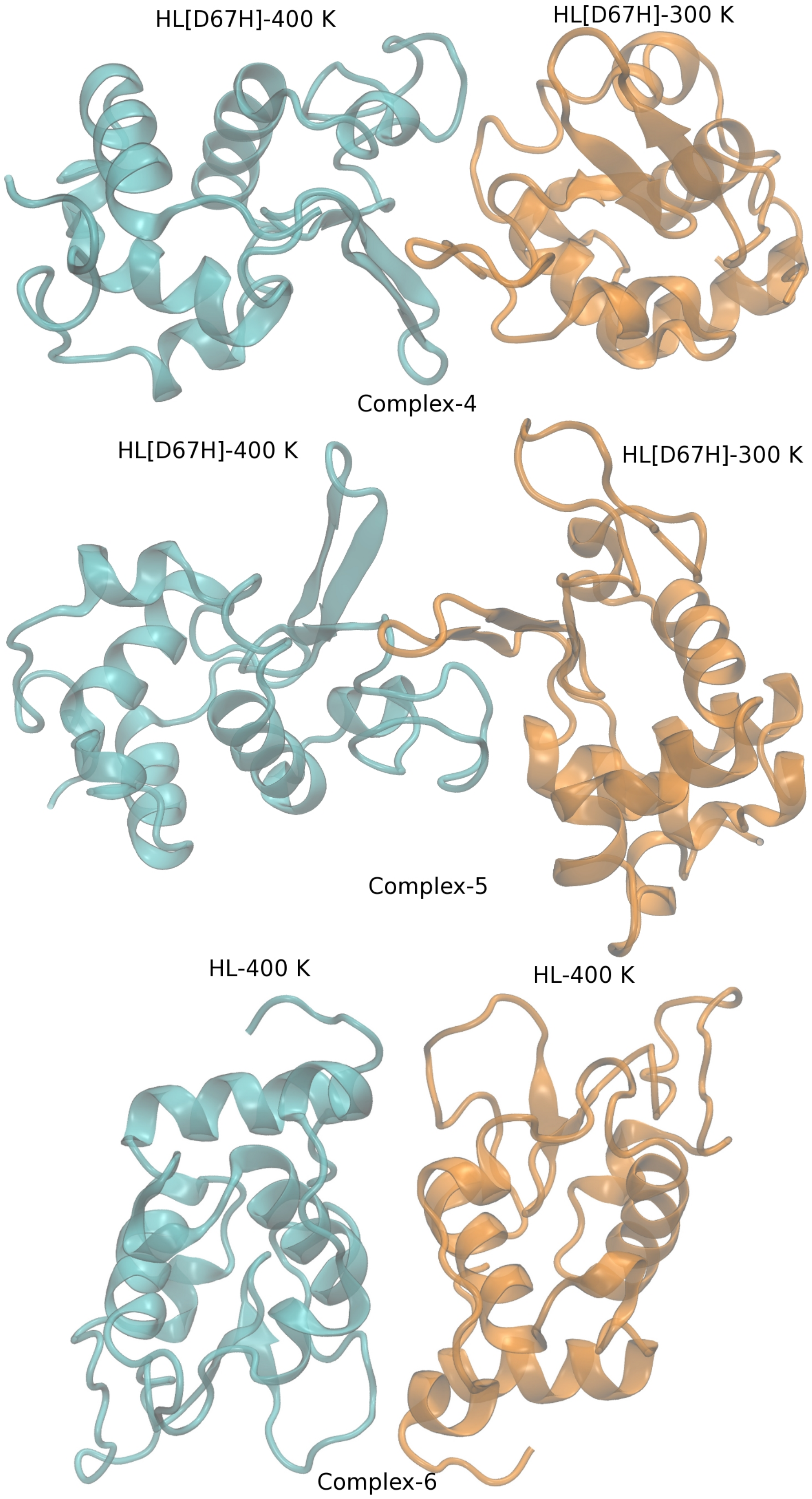

Figure D: Binding modes of HL[D67H] dimers obtained from blind docking of the structure at 400 K with a structure at 300 K (complex-4 and 5). Binding mode of HL dimer obtained from blind docking of the structures at 400 K (complex-6).

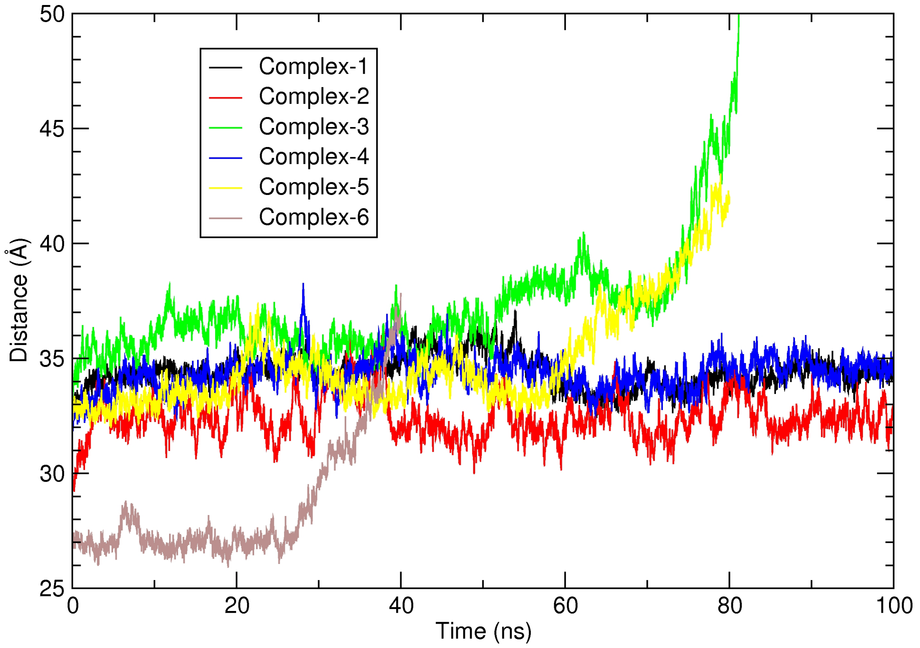

Figure E: Time series of the center of mass to center of mass distance between the two monomers obtained from the MD simulations of the six complexes. Complex-1, 2, and 4 remain stably bound whereas Complex-3, 5, and 6 dissociate.

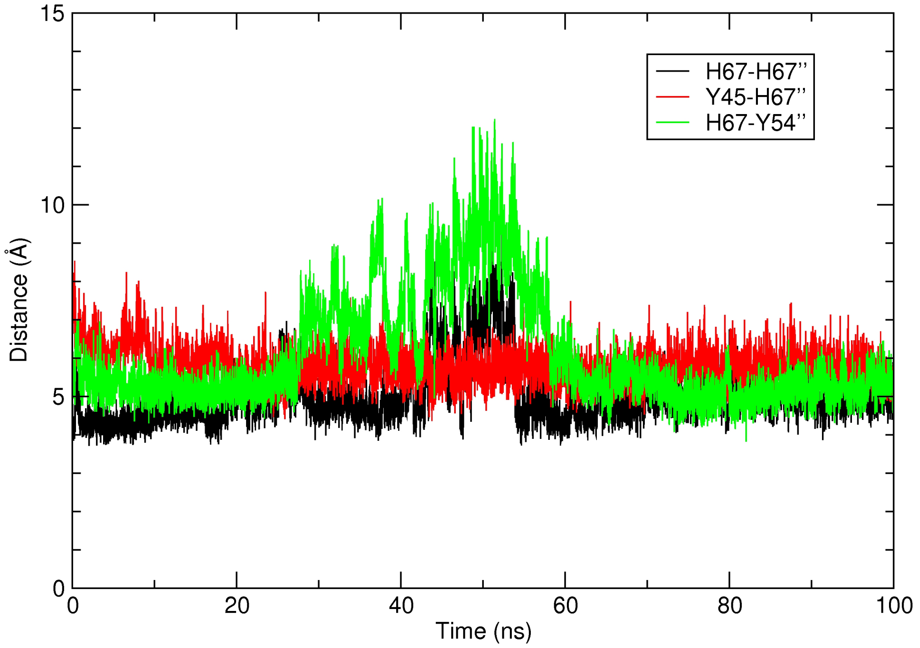

Figure F: Time series of the distances between the centers of the aromatic rings for the pi-stacking interactions between H67–H67″, Y45–H67″, and H67–Y54″ in Complex-1.
